# Using Lidar Data to Analyse Sinkhole Characteristics Relevant for Understory Vegetation under Forest Cover—Case Study of a High Karst Area in the Dinaric Mountains

**DOI:** 10.1371/journal.pone.0122070

**Published:** 2015-03-20

**Authors:** Milan Kobal, Irena Bertoncelj, Francesco Pirotti, Igor Dakskobler, Lado Kutnar

**Affiliations:** 1 Biotechnical Faculty, University in Ljubljana, Department of Forestry and Renewable Forest Resources, Ljubljana, Slovenia; 2 National Institute of Biology, Department of Freshwater and Terrestrial Ecosystems Research, Ljubljana, Slovenia; 3 University of Padova, Interdepartmental Research Center in Geomatics, Department of Land, Environment, Agriculture and Forestry, Padova, Italy; 4 Research Centre of the Slovenian Academy of Sciences and Arts, Jovan Hadži Institute of Biology, Ljubljana, Slovenia; NASA Marshall Space Flight Center, UNITED STATES

## Abstract

In this article, we investigate the potential for detection and characterization of sinkholes under dense forest cover by using airborne laser scanning data. Laser pulse returns from the ground provide important data for the estimation of digital elevation model (DEM), which can be used for further processing. The main objectives of this study were to map and determine the geomorphometric characteristics of a large number of sinkholes and to investigate the correlations between geomorphology and vegetation in areas with such characteristics. The selected study area has very low anthropogenic influences and is particularly suitable for studying undisturbed karst sinkholes. The information extracted from this study regarding the shapes and depths of sinkholes show significant directionality for both orientation of sinkholes and their distribution over the area. Furthermore, significant differences in vegetation diversity and composition occur inside and outside the sinkholes, which indicates their presence has important ecological impacts.

## Introduction

Sinkholes, which are also described as dolines, are depressions in terrain that represent a unique feature of karst landscapes with which several impacts and hazards can be associated [1,2]. Sinkholes were the first type of karst landform that were subjected to morphometric analysis [3]. Early studies [4–6] indicated that a large variety of morphometric parameters exist among sinkholes, mainly depending on karst development and sinkhole genesis.

Sinkhole genesis is related to different processes and cannot be simplified into one common model. Corrosion, collapse, and climatic models, or combinations of these models, have been used to explain the genesis of different sinkholes [7]. Corrosion is an essential process in all of these models [7,8]. A descriptive-genetic classification of eight basic types of sinkholes was developed based on the following geological structure elements: (i) bed locations (ii) degree of rock fracture, and (iii) morphological properties of the sinkholes [7]. The eight basic types of sinkholes include stratification sinkholes, fissure sinkholes, bedded-fissured sinkholes, broken sinkholes, near-fault sinkholes, fault sinkholes, contact sinkholes, and reproduced sinkholes [7]. Although pure types of sinkholes are rare in nature, combinations of them are more common.

Traditionally, morphometric studies of karst landscapes are based on topographic maps and air photographs [9,10], from which digital elevation models (DEM) are derived using different photogrammetric methods. Here, we refer to DEM in the sense of height information of the bare ground plane as by [11]. The greatest disadvantages of such DEM are their insufficient resolution and accuracy, especially in forested karst areas [12]. Consequently, only costly and time-consuming fieldwork and cave surveys can provide the data necessary for performing morphometric studies of karst landforms [3].

Active and passive remote sensing techniques have been tested for detecting karst depressions [13, 14]. When using these techniques, vegetation obstruction can significantly limit the information obtained from the ground surface. Airborne laser scanning has proven in several studies to be able to penetrate dense forest canopies and display the underlying topography of the ground [15–17]. Due to this unique ability, high-density airborne laser scanning data have been used for many applications to obtain high-resolution topographic profiles of the bare ground.

For example, in forestry, lidar data are used to estimate biomass [18], to survey the 3D structure of the forest [19] and detect stem positions [20]. In addition, the full-waveform information from the return pulse has been used to improve these results [21]. This technique is also commonly used in hydrology [22] and soil studies [23]. Geomorphology studies in areas where vegetation adds noise to the laser dataset require careful filtering to discriminate between vegetation and the ground [24–27], especially for landslide monitoring [28]. However, studies aimed at automatically detecting sinkhole characteristics from lidar-based digital elevation models are not common. The use of lidar for 3D characterisation of sinkholes was applied in a study conducted near the Dead Sea, in which ArcHydro module was used to automatically delineate sinkholes [29]. The importance of pre-processing the DEM to guarantee that it is “hydrologically correct” for successive analyses was discussed by Doctor and Young [30]. Image processing techniques (erosion, fitting and pruning operators) were used by [31] to automatically detect sinkholes, who identified 97 true positives (correct sinkhole detection), 21 false positives (sinkholes were detected but did not exists) and 9 false negatives (sinkholes existed but were not detected). However, none of these methods have been tested in the presence of forest tree-canopies. Tree canopies can decrease the homogeneity of the spatial distribution obtained from ground hits. This effect depends on the distribution of the canopy density, especially where high-resolution surveys are conducted because higher emitter frequencies correspond with lower laser pulse energies and lower penetration capabilities [32].

Information regarding the locations and characteristics of sinkholes is important in several aspects. In some areas sinkholes are associated with environmental and engineering problems [1]. On the other hand, sinkholes have been proven valuable because of their specific habitat features and their effects on local biodiversity [33]. The microclimates in sinkholes range from humid and cool conditions on the bottom to warmer and drier conditions on the slopes, which are reflected by the plant species composition [33–36]. Consequently sinkholes can act as refugia for mountain species [33] and can increase local biodiversity. Furthermore, sinkhole characteristics affect vegetation patterns, with larger sinkholes having more pronounced vegetation inversions and providing habitat for a greater number of vascular plant species [37].

High karst areas in Slovenia are mainly covered by Dinaric silver fir—European beech forests (*Omphalodo-Fagetum*), which are one of the most extensive forest communities within the Natura 2000 network in south-eastern Europe and one of the most widespread types of forests in Slovenia [38]. These forests significantly add to the overall plant species diversity of the 91K0—Illyrian *Fagus sylvatica* (*Aremonio-Fagion*) habitat type of Natura 2000 [39]. Because the total area of sinkholes in this karst landscape may be relatively large, their influences on ecological processes and their contributions to the biodiversity of Dinaric silver fir—European beech forests are considerable. Stands of some subunits of the *Omphalodo-Fagetum* (*aceretosum pseudoplatani*, *stellarietosum montanae*) association generally thrive in the bottom and on the flanks of small sinkholes, as well as on foothills and the lower edges of larger sinkholes [40,41].

In this paper, we present a case study of mapping sinkholes and calculating their characteristics under a dense forest tree-canopy by leveraging lidar data to obtain a high-resolution digital elevation model. The objective of this study was to calculate and describe the morphometric characteristics of a large number of sinkholes in diverse karst terrains under dense forest tree-canopies using a lidar-derived DEM. In contrast with traditional field mapping methods, this method is cheaper, faster, and safer regarding the collection of ground measurements from field-campaigns in areas with low accessibility due to irregular morphology and the presence of sinkholes. We used the obtained sinkhole locations and characteristics to compare the plant species richness and compositions between plots inside and outside of sinkholes. The selected study area is in Leskova dolina in Slovenia, an area with very low anthropogenic influence that is particularly suitable for studying the characteristics of undisturbed karst sinkholes and their effects on the plant community.

## Methods

### 2.1 Ethics statement

Airborne laser scanning was conducted in state forests where no permits were required. Plant species diversity assessments were carried out in accordance with legal and environmental requirements and with the ethical codes or norms of the community and country in which the activities occurred. Plant species assessments in the field were conducted in a non-destructive way, and no protected or rare species were removed from the field.

### 2.2 Study area

**Fig 1:**
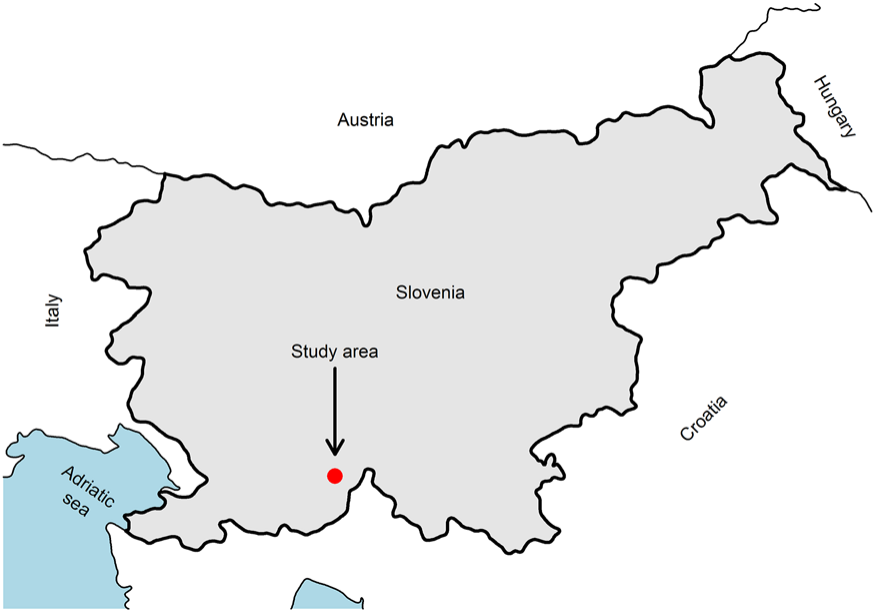
Locations of the Leskova dolina study area in Slovenia.

We identified and mapped sinkholes within a predominantly forested Leskova dolina landscape covering an area of 5212.2 ha in the Dinaric Mountains of southwest Slovenia (the centre of the area is approximately located at Longitude = 14.46° and Latitude = 45.62° in the WGS84 datum, Fig. 1). The karst geology at the site is characterised by numerous sinkholes and limestone outcrops, which resulted from very diverse micro-topography. The soils in the area were predominantly Lithosols, Leptosols, Cambisols and Luvisols and were predominantly derived from limestone and dolomite parent materials. The soil depth varies from 0 to 300 cm, depending on the micro topographic position. Precipitation is evenly distributed throughout the year, with a mean annual precipitation of 2150 mm. The mean temperature is 6.5°C, and late spring and early autumn frosts are common.

Forests cover 97.6% of the study area, with the prevalent vegetation community consisting of Dinaric silver fir—European beech forest (*Omphalodo-Fagetum*), with European beech (*Fagus sylvatica*), silver fir (*Abies alba*), and Norway spruce (*Picea abies*) as the dominant tree species. The shape, size and distribution significantly affect the forest soil, hydrological characteristics [42–44], and, consequently, the tree growth [45]. Under such karstic conditions with a high number of sinkholes, forest management practices must be adapted to very rough and sensitive terrain.

### 2.3 Airborne laser scanning

Lidar data were acquired by a private company using a Eurocopter EC 120B helicopter at a relative flight height of 400 to 600 m and with full-waveform laser scanner (Riegl LM5600) using laser impulses at a frequency of 180 kHz. The surface point accuracy, excluding the GPS errors, was 10 cm in the horizontal plane and 3 cm in the vertical plane. The trajectory and orientation of the helicopter were determined using Novatel OEV/OEM4 GPS recording measurements (at 10 Hz frequency) and an INS IMU-IIe unit based on Fibre-Optic Gyros. The point density was 30 points m^-2^, with an average footprint of 30 cm. Scanning was conducted during October 2009, and data were processed using a Microstation v2004 (Bentley) with Terrasolid packages.

To classify the point cloud, we applied a procedure to remove outlier points. Then, we used the Axellson’s iterative triangulation method [46] to assess which points could be considered as belonging to the ground plane. A filtering algorithm is based on triangular irregular network TIN; firstly a sparse TIN is created from the seed points which are later densified in an iterative process. The TIN adapts to the data points from below and new points are added only if they meet certain data derived threshold parameters. At the end, all points are classified as either ground or object. For details see [46]. This step is particularly important because the parameters used in the module must maximise the number of true positives in the ground class (ground points correctly classified) and minimise the number of false negatives (ground points which are classified as not-ground) and false positives (vegetation points classified as ground). Although this step seems trivial, it is particularly critical in this case study because the micro-topography requires careful tuning of the parameters, depending on the complexity and scale of the ground surface elements, to successfully remove the effects of the forest canopy without significantly affecting the topographic details. Meng et al. [47] provide a more detailed critical analysis regarding this aspect. Next, ground points were used to create a DEM in raster format with a cell size of 1 × 1 m for an effective study area of 5212.2 ha.

### 2.4 Sinkhole extraction

**Fig 2:**
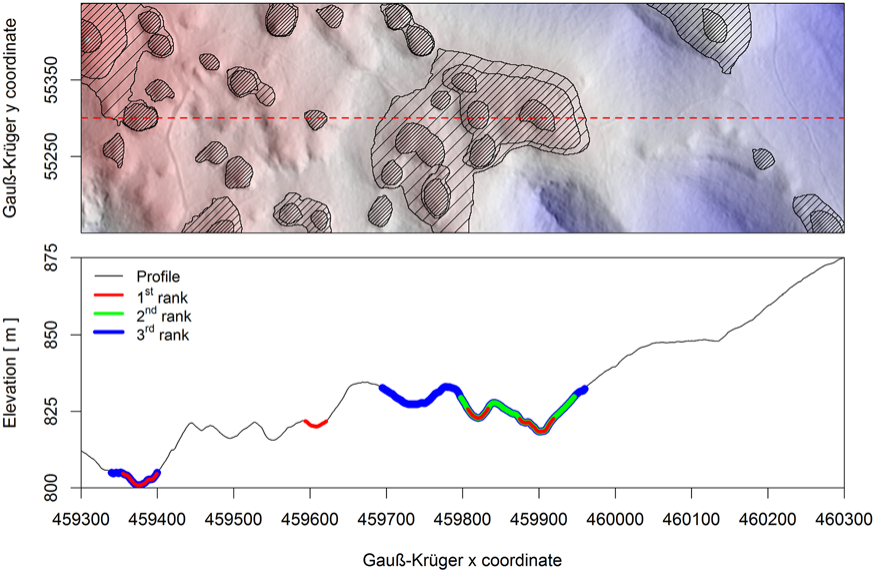
Hill shaded relief (above) of a section of the study area with 1st rank (grey), 2nd rank (narrow stripes) and 3rd rank (broad stripes) sinkholes. The lidar-based profile of the cross section is shown in the bottom graph and is marked with a dotted line on the topographic map, which shows all three ranks of sinkholes.

We extracted sinkholes using the algorithm described by [48], which is based on the information from a DEM. This algorithm is based on water flow simulations on a surface DEM and incorporates four steps: (i) watershed delineation, (ii) confining sinkholes, (iii) confining higher rank sinkholes and (iv) extracting non-karstic sinkholes. The watershed delineation step uses GIS reconditioning of the lidar DEM for watershed analysis that employs the ArcHydro tools in ArcMap 10.2, as described by Doctor and Young [30]. To correctly model surface flow patterns, the areas of closed depressions were filled to their hydrologic spill point, the flow direction for each cell was identified, and each cell received a value representing the total number of cells that drained into it [49]. In the second step, effluent levels confined the sinkholes, and the cells below the effluent level were designated as part of the sinkhole. The input data in this step included a layer containing delineated watersheds and a layer containing elevation information. For each delineated watershed, the cell with the lowest elevation among the watershed boundary cells was defined as the effluent level. The output consisted of a list of watersheds with elevation information for the effluent points, and the points below the effluent level were assigned as forming a sinkhole. In the third step, sinkholes were ranked according to their locations and sizes with respect to surrounding sinkholes. The 1^st^ sinkholes are the smallest and are located within larger sinkholes of a higher rank [48]. Fig. 2 contains a graphical presentation of sinkholes of different ranks. When a smaller sinkhole was located within a larger sinkhole, the effluent level was first determined for the smaller sinkhole. After filling in the smaller sinkhole, steps 1–3 were repeated to delineate a larger sinkhole, which was assigned a higher rank. This procedure was repeated until all of the depressions were filled in and the water flow was equivalent at all points.

Finally, the non-karstic sinkholes, which can be errors in the DEM generation process, [50] were extracted. In the published literature, karst solution sinkholes are described as basins that are more than 2 m deep and more than 10 m in diameter [51–53]. Using both criterions, we eliminated non-karstic sinkholes.

### 2.5 Calculation of sinkhole characteristics

**Fig 3:**
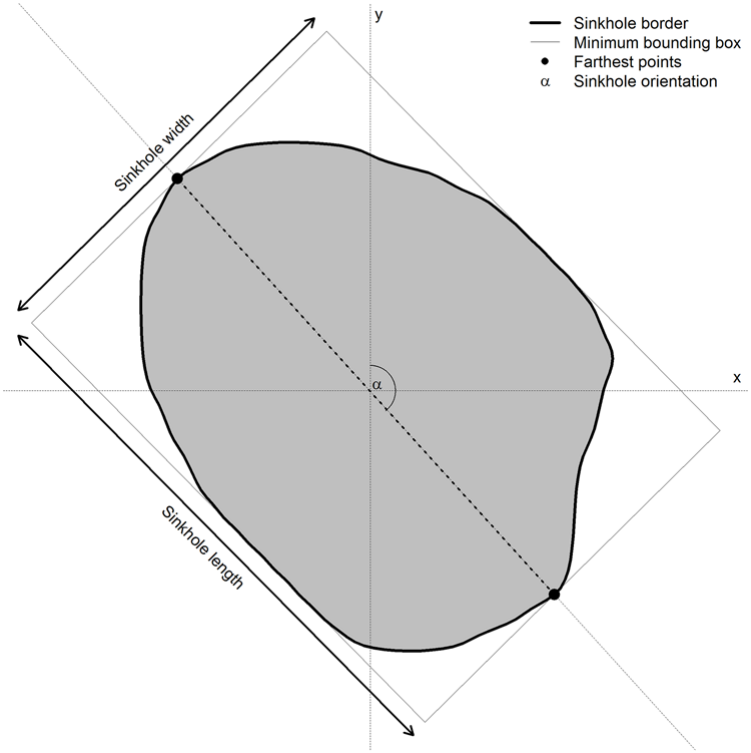
Measurements used to estimate the sinkhole geometry.

The extracted karst sinkholes were vectorised from the raster cells, and the basic morphometric characteristics: (i) width, (ii) length, (iii) area, (iv) depth, (v) volume and (vi) orientation were calculated for each sinkhole of each rank. The rotating callipers method [54] was used to delineate the minimum bounding box for a set of points and define a convex polygon for each sinkhole. The sinkhole length was defined as the length of the major axis and the sinkhole width was defined as the length of the minor axis (Fig. 3). The volume of each sinkhole was calculated as the sum of the differences between the maximum elevation within the sinkhole and the elevation of each cell of a DEM within a sinkhole. The orientation was calculated as an azimuth of the line connecting the two farthest points within the sinkhole (Fig. 3).

The elongation ratio (*R*_*e*_) was originally developed for drainage system studies by [55] and was defined as the ratio between the diameter of a circle with the same area and the diameter of the basin to the maximum basin length. However, in the morphometric analysis of sinkholes, the elongation ratio *R*_*e*_ is the ratio between the major and the minor axes [4, 10, 56]. In this study, we calculated the elongation ratio (Equation 1) as:

$$R_{e}=\frac{S_{length}}{S_{width}}$$ (1)

where *S*_*length*_ and *S*_*width*_ are the measured length and width of a sinkhole, respectively.

According to [3], we classified the sinkholes into 4 groups regarding their elongation ratios, *R*_*e*_: (i) circular and sub-circular (*R*_*e*_ ≤ 1.21), (ii) elliptical (1.21 < *R*_*e*_ ≤ 1.65), (iii) sub-elliptical (1.65 < *R*_*e*_ ≤ 1.8) and (iv) elongated (*R*_*e*_ > 1.8).

Regarding the circularity indexes of sinkholes, no common method of calculation exists [30, 57, 58]. However, its definition is clear, it is the ratio between the measured sinkhole area and the area of a circle with the same perimeter; thus, a measure of the deviation of a polygon from a perfect circle (the circularity index of a perfect circle is equal to 1). In this study, we calculated the circularity index (Equation 2) as follows:

$$Circ_{i}=\frac{A_{m}}{\pi \cdot {\left(2\cdot \frac{A_{m}}{P_{m}}\right)}^{2}}$$ (2)

where *A*_*m*_ and *P*_*m*_ are the measured area and perimeter of a sinkhole, respectively.

To show where the sinkholes were concentrated, we calculated the surface density. For this calculation, we used the kernel density, where the resulting surfaces surrounding each pixel in the DEM are based on a quadratic formula, with the highest value at the centre of the surface (the point location) and tapering to zero at the search radius distance. For each output cell, the total number of the accumulated intersections of the individual spread surfaces was calculated. Only the density map of the first ranking sinkholes was calculated, and the search radius within each density was set to 564.2 m (radii for area of 1 km^2^).

### 2.6 Vegetation sampling

In a 20 ha sub-section of the study area, 65 circular sampling plots were surveyed in a 50 × 50 m grid. In each plot, a tree stem was selected, and an area with a radius of eight meters was delineated. In this area, all the plant species in the herb, fern and moss layers and their frequency were recorded using the Central European method, following the Braun—Blanquet approach [59]. Based on a field assessment of the plant species, the Shannon (H', Equation 3) and Simpson (D, Equation 4) diversity Indexes were calculated as follows:

$$H'=-\sum_{n=1}^{i}p_{i}\cdot ln(p_{i})$$ (3)

$$D=1-\sum_{n=1}^{i}p_{i}^{2}$$ (4)

where *p*_*i*_ is proportional to the surface area of a certain plant species.

### 2.7 Statistical analysis and model selection

All sinkhole characteristic analyses were carried out in R programming language, version 3.0.2 [60], using our own scripts and the following available packages: sp [61,62], raster [63], maptools [64], igraph [65] and vegan [66]. Levene's test was used to assess the equality of variances for the *R*_*e*_, *Circ*_*i*_, species richness and diversity indices. Due to the occurrence of non-homogeneous variances (Levene’s test, p < 0.05), the elongation ration (*R*_*e*_) and circularity index (*Circ*_*i*_) were compared between sinkholes with different ranks by using a non-parametric Kruskal-Wallis test and a multiple comparison test after Kruskal-Wallis [67] in the “pgirmess” R library [68]. The species richness and diversity indices (Simpson and Shannon) inside and outside (n = 35) the sinkholes were compared using a Student’s *t*-test (Levene’s test, p > 0.05). The multiple regression routine for the log-transformed data of sinkhole depth, area and sinkhole volume (due to high skewness) was used to relate a sinkhole’s depth and area with its volume. Models were compared using partial F-tests and the Akaike’s Information Criterion (AIC). The following two main factors were considered: i) position of the plot: inside vs. outside the sinkhole, and ii) the type of parent material: dolomite vs. limestone. The differences among the plant species compositions of the plots were extracted using a detrended correspondence analysis DCA [69].

## Results

**Fig 4:**
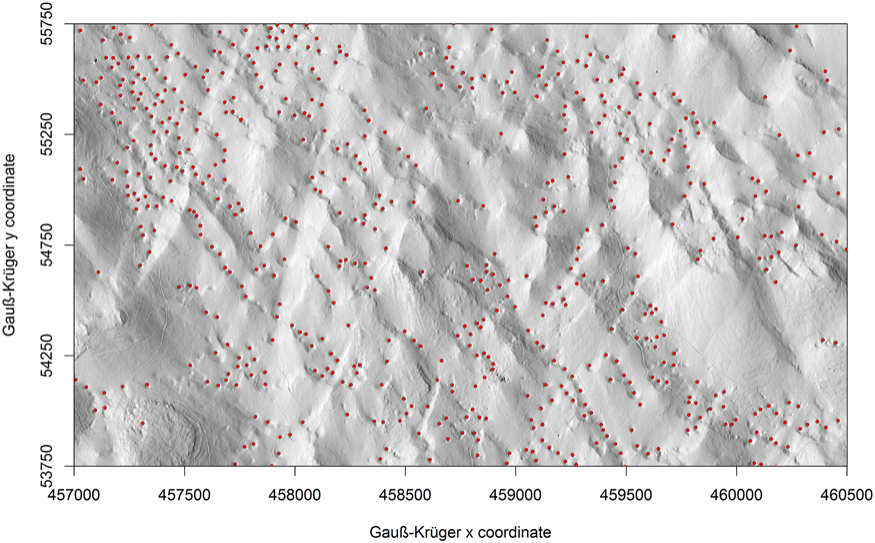
Map of the 1st ranking sinkholes (dots) in the Leskova dolina study area and the eight sinkhole density classes [km-2] (colour bands). The search radius was set to 564.2 m (radii for area of 1 km^2^).

Overall, 2660 sinkholes were detected within our study area using the lidar-derived DEM with a cell size of 1 × 1 m. Most of the sinkholes (2095) were ranked 1^st^ (40.2 km^-2^), followed by 473 sinkholes ranked 2^nd^ (9.1 km^-2^), 79 ranked 3^rd^ (1.5 km^-2^), 12 ranked 4^th^ rank (0.25 km^-2^), and one ranked 5^th^ (0.02 km^-2^). The density of 1^st^ ranking sinkholes varied considerably within the study area (Fig. 4) with overall density of 40.2 sinkholes per km^2^. Sinkholes have an aggregate area of 1.7 km, representing about 3.3% of the Leskova dolina (Table 1). Half of the Leskova dolina area (26.1 km) had the lowest sinkhole density of 15.7 sinkholes per km^2^ (Table 1). The highest density of 1^st^ ranking sinkholes was 165.1 per km^2^ in an area of 0.6 km (1.2%) of the area of Leskova dolina. Only 0.3% of study area did not contain any sinkholes.

**Table 1:** The total number of 1^**st**^ ranking sinkholes, their density and the area they cover in six density level classes in Leskova dolina study area shown in Fig. 4.

Density	Total area[km^2^]	Total area[%]	Number ofsinkholes	Sinkhole density[km^-2^]	Area of sinkholes[km^2^]	Area of sinkholes[%]
0	0.15	0.3	0	0.0	0.00	0.00
1–25	26.09	50.0	410	15.7	0.43	1.63
26–50	10.66	20.3	359	33.7	0.33	3.13
51–75	7.89	15.1	524	66.4	0.41	5.15
76–100	5.19	9.9	474	91.3	0.30	5.73
101–125	1.68	3.2	224	133.3	0.16	9.23
126–150	0.63	1.2	104	165.1	0.08	12.31
Sum	52.14	100.0	2095	40.2	1.70	3.25

**Fig 5:**
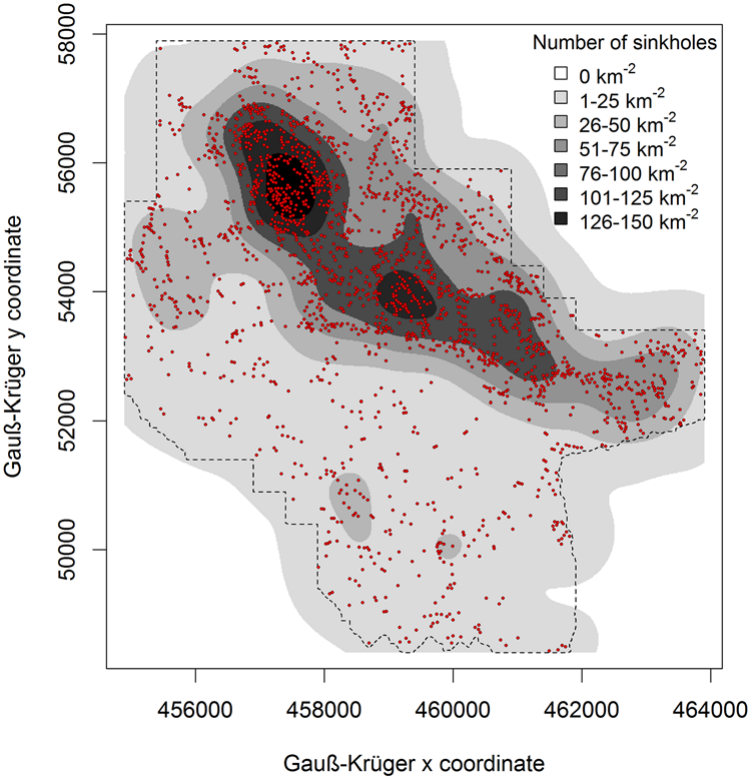
Hill-shaded relief with the spatial distributions of the 1st ranking sinkholes in a portion of the Leskova dolina study area.

The percentages of the areas covered by the sinkholes ranked 1^st^, 2^nd^, 3^rd^, 4^th^ and 5^th^ in the study area were 3.3%, 4.3%, 5.7%, 3.7% and 3.1%, respectively. An example of the sinkhole position is shown in Fig. 5. Majority of 1^st^ ranking sinkholes were located between 687.6 m a.s.l and 1011.5 m a.s.l. Only 10% of 1^st^ rank sinkholes are located in elevation between 1011.5 m a.s.l and 1552.5 m a.s.l.

**Fig 6:**
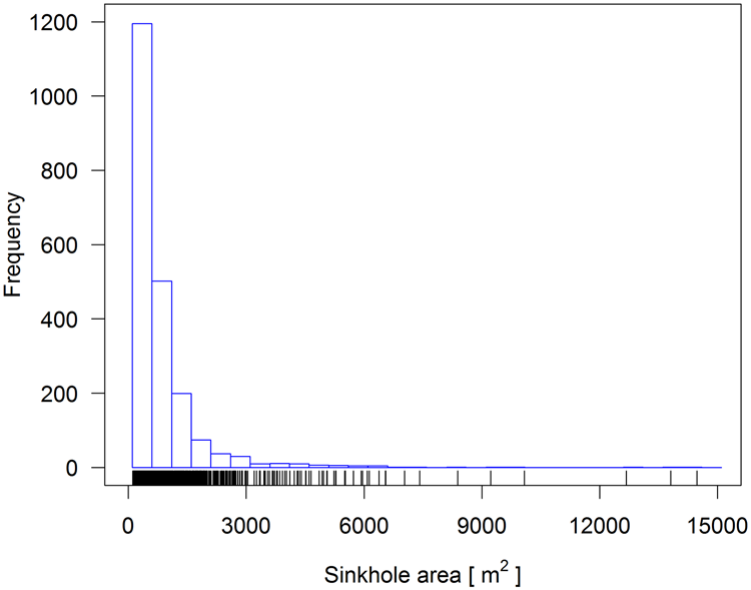
Frequency distribution of the 1^st^ ranking sinkhole area in the Leskova dolina study area.

The morphometric characteristics of the 1^st^, 2^nd^, 3^rd^ and 4^th^ ranking sinkholes in the Leskova dolina study area are given in Table 2. The mean width, length, depth, area and volume of the sinkholes increased with their rank. The maximum sinkhole depth ranged from 39.2 (1^st^ rank) to 48.4 m (4^th^ rank), and only the sinkhole ranked 5^th^ (not shown in Table 2) reached a depth of 52.8 m. The width of the 5^th^ ranking sinkhole was 1317.9 m, the length was 2418.5 m, the total area was 131.05 ha and the total volume was approximately 22 million cubic meters. The distribution area of the 1^st^ ranking sinkholes roughly resembled a negative exponential, with 57% of sinkholes below 600 m^2^. However, larger sinkholes (i.e., above 5000 m^2^) were relatively abundant (Fig. 6). Similar size distributions were also evident for other sinkhole ranks, except for the single sinkhole in the 5^th^ rank and the low number of sinkholes in the 4^th^ rank, which did not allow us to determine the shapes of their distributions.

**Table 2:** Median, quartile, minimum and maximum values of width, length, orientation, depth, area and volume for the sinkholes of four different ranks.

Rank	Summary	Width [m]	Length [m]	Orientation [°]	Depth [m]	Area [m^2^]	Volume [m^3^]
1^st^ rank (n = 2095)	Min	10.4	11.4	0	2.0	108.2	89.0
	1^st^ Qu	17.9	22.8	56	2.7	315.2	370.2
	Median	23.3	29.3	93	3.7	530.6	735.9
	Mean	26.1	33.8	93	4.4	809.5	1,957.6
	3^rd^ Qu	30.7	39.3	130	5.1	903.6	1,605.3
	Max	129.8	195.2	180	39.2	14,475.6	102,488.4
2^nd^ rank (n = 473)	Min	15.1	18.4	0	2.2	214.2	186.1
	1^st^ Qu	32.5	55.4	65	4.8	1,295.9	2,203.4
	Median	47.9	78.6	106	6.7	2,445.2	5,492.6
	Mean	57.4	93.0	99	8.1	4,694.2	24,568.6
	3^rd^ Qu	68.1	115.4	137	9.3	4,949.4	13,150.9
	Max	394.3	435.0	180	66.6	114,903.1	2,383,237.6
3^rd^ rank (n = 79)	Min	23.8	33.3	3	4.4	497.8	668.3
	1^st^ Qu	74.4	147.5	76	8.6	7,370.4	23,886.5
	Median	115.0	220.0	108	12.3	13,878.1	41,431.9
	Mean	148.2	295.0	100	14.5	37,447.9	328,792.9
	3^rd^ Qu	176.7	328.9	135	18.1	36,848.9	163,260.2
	Max	676.8	1,470.6	172	46.0	354,649.0	6,124,113.8
4^th^ rank (n = 12)	Min	58.6	181.9	2	8.7	5030.3	14,875.1
	1^st^ Qu	198.2	233.4	45	19.7	22,300.5	83,826.5
	Median	293.0	477.0	91	24.4	63,257.1	440,861.5
	Mean	367.6	576.0	78	25.9	148,278.1	1,519,267.6
	3^rd^ Qu	457.7	770.8	115	34.6	208,367.2	2,870,008.7
	Max	942.9	1,279.1	143	48.8	515,923.2	4,661,818.9

**Fig 7:**
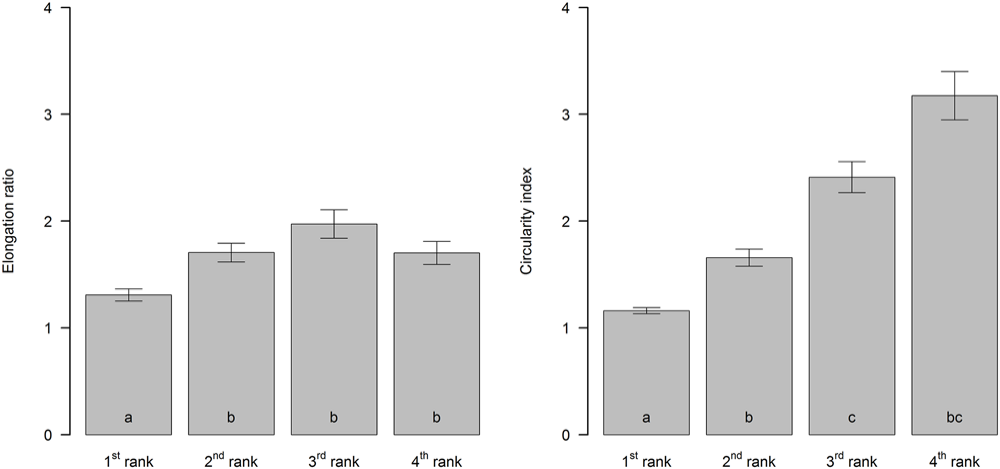
Elongation ratios Re and circularity indexes Circi for the four sinkhole ranks in the Leskova dolina study area. The bar plots marked with the same letters were not significantly different according to the Kruskal-Wallis multiple comparison test.

According to [3], the classification results of sinkholes according to the elongation ratio identified 39.8% of the sinkholes as circular, 41.1% as elliptical and 13.5% as elongated (*R*_*e*_ above 1.8). The shapes of the sinkholes also varied according to their rank. The sinkholes ranked 1^st^ were significantly more circular than those ranked 2^nd^, 3^rd^ or 4^th^ (Fig. 7; Kruskal-Wallis χ^2^ = 466.8; p < 0.001). Similarly, the higher ranked sinkholes were significantly less circular according to their circularity index, with a gradual decrease in circularity from the 1^st^ to 3^rd^ rank (Fig. 7; Kruskal-Wallis χ^2^ = 1082.9; p < 0.001). Twelve sinkholes that were ranked 4^th^ had similar values to those of the 2^nd^ and 3^rd^ ranks (Fig. 7). The single sinkhole ranked 5^th^ was omitted from this comparison.

A strong linear correlation was observed between the sinkhole area and the sinkhole volume (Table 3). Sinkhole depths explain 75% of variability in sinkhole volume (model M1, Table 3). The sinkhole area explained 95% of the variation of the sinkhole volume (model M2, Table 3). The prediction power was further improved by model M3, in which sinkhole depth and sinkhole area were both used as explanatory variables in the model (Table 3). A comparison between the M3 model and the previous two models (M1 and M2) with partial F-tests suggested that the nested model was significantly better (p < 0.001).

**Table 3:** Rankings of the three models based on their abilities to predict sinkhole volume (*S*_*vol*_) from sinkhole depth (*S*_*depth*_) and sinkhole area (*S*_*area*_) using Akaike’s information criterion and the results of the regression for each model.

Name	Model	SE	Adjusted *R* ^*2*^	AIC
M1	ln(S_vol_) = 3.37 + 2.56 × ln(S_depth_)	0.74	0.79	5930.1
M2	ln(S_vol_) = -1.58 + 1.31 × ln(S_area_)	0.35	0.95	2036.2
M3	ln(S_vol_) = -0.69 + 0.92 × ln(S_depth_) + 0.97 × ln(S_area_)	0.16	0.99	-2297.9

**Fig 8:**
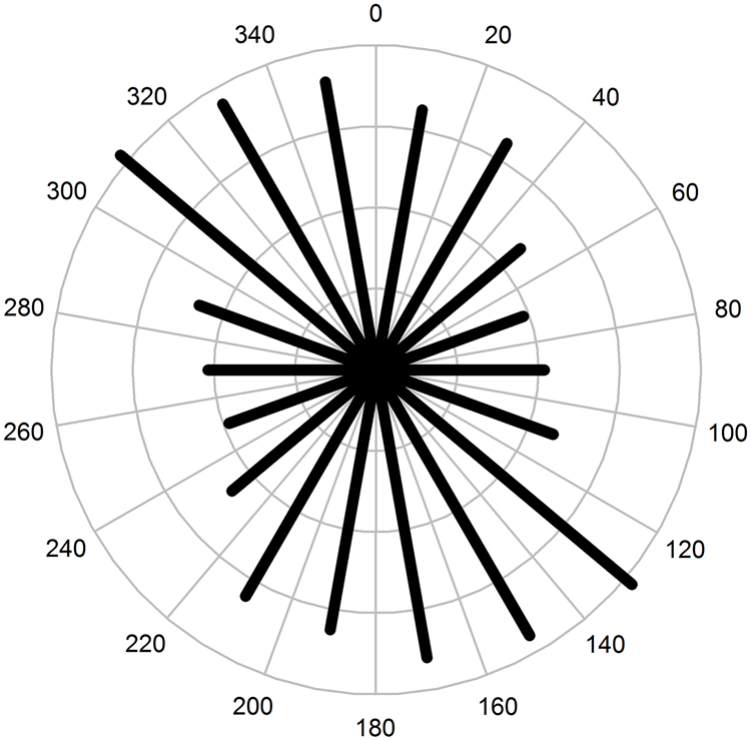
Frequency distribution of sinkhole orientation for 2660 sinkholes in the Leskova dolina study area.

All of the sinkholes, regardless of their elongation ratio, were included in the orientation study, which potentially blurred the karstification effects and subsidence processes. Examination of the sinkhole orientations indicated a strong influence of the NW—SE jointing (Fig. 8). Two peaks of sinkhole orientation were observed between 10 and 30° of the azimuth, and a higher peak was observed between 130 and 170° (Fig. 8). General orientation is also evident from the hill-shaded relief shown in Fig. 5.

In the vegetation survey, 115 vascular plant species, 17 fern species and 27 moss and lichen species were identified. The plots inside the sinkholes had significantly higher numbers of all plant species (Student’s *t*-test = -2.19; p < 0.05) and vascular plant species (Student’s *t*-test = -2.19; p < 0.05). The species richness of mosses (Student’s *t*-test = 0.59; p > 0.05) and ferns (Student’s *t*-test = -0.64; p > 0.05) did not differ between the plots located inside and outside of the sinkholes (Table 4). The calculated values of the Simpson diversity index (Student’s *t*-test = -2.66; p < 0.05) and Shannon diversity index (Student’s *t*-test = -2.92; p < 0.05) of the plant species were significantly higher in the plots inside the sinkholes relative to those outside the sinkholes (Table 4).

**Table 4:** Comparison of plant species richness and two diversity indices (± 95 confidence interval) of four groups (mosses, ferns, seed plants, all plants) in 65 plots inside (n = 30) and outside (n = 35) of sinkholes.

Variable	Inside the sinkhole		Outside the sinkhole		OverallTotal
	Mean	Total	Mean	Total	
Number of moss species	8.6 ± 0.9^a^	26	9.0 ± 0.9^a^	24	27
Number of fern species	5.3 ± 1.0^a^	17	4.8 ± 0.8^a^	13	17
Number of seed plant species	29.8 ± 2.3^a^	94	26.6 ± 1.8^b^	88	115
Number of all plant species	43.7 ± 2.1^a^	137	40.4 ± 2.1^b^	125	159
Simpson diversity index	0.89 ± 0.02^a^		0.85 ± 0.02^b^		
Shannon diversity index	3.00 ± 0.13^a^		2.72 ± 0.14^b^		

The number marked with the same letters were not significantly different according to the Kruskal-Wallis multiple comparison test (species richness) and t-test (diversity indices).

**Fig 9:**
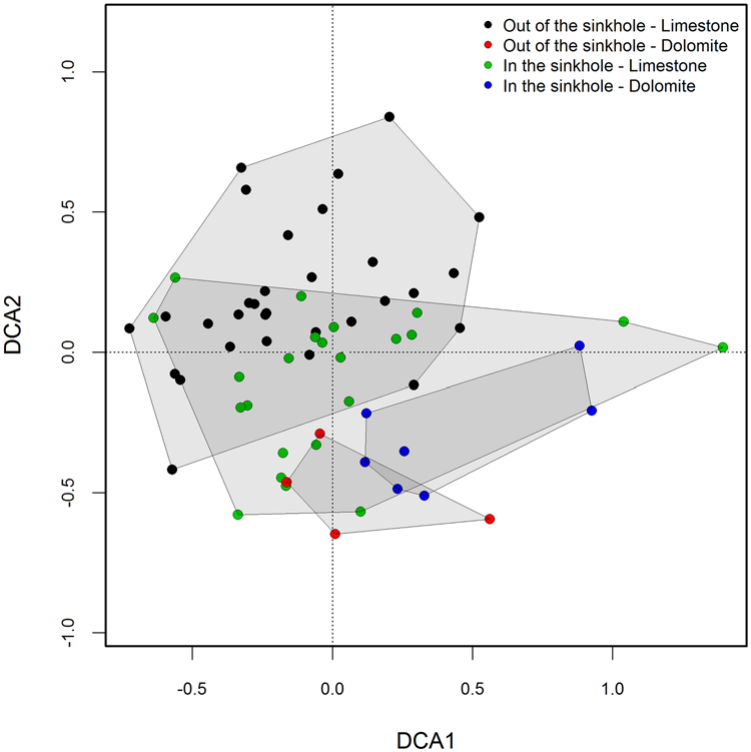
The detrended correspondence analysis (DCA) of plant community composition at 65 sampling plots in the Leskova dolina study area. The sample scores of 65 surveyed plots on the first two axes are grouped according to their location (outside vs inside the sinkhole) and parent material (limestone vs dolomite).

The DCA of the plant community composition clearly separated the plots located on limestone in the upper part of the biplot from those on dolomite located in the lower part of the biplot (Fig. 9). The DCA did not distinguish between the plots according to their locations inside or outside of the sinkholes. However, the community composition in the sampling plots inside the sinkholes was less affected by the parent material (Fig. 9).

## Discussion

The Dinaric Mountains cover a large area of the Balkan Peninsula with different altitudes, climatic conditions and geologic compositions. Therefore, it is not surprising that a large variety of sinkholes (in terms of shape and size) exist. For example, in the Classical Karst (at the boundary between NE Italy and Slovenia), the most common planimetric shape was circular, and most of the examined features corresponded with solution sinkholes [70]. Our results only confirmed this for the sinkholes that were ranked 1^st^. However, the difference of methods for sinkhole detection did not allow us to directly compare these results. Contrary to the majority of existing methods for sinkhole extraction methods used in our study enabled us extraction of sinkholes belonging to different ranks. Only circularity of sinkholes ranked 1^st^ and 2^nd^ was within the same range as sinkholes in Florida [71] and in Spain [72]. The sinkholes with higher ranks were increasingly less circular.

The sinkhole density differed greatly within our study area of Leskova dolina. Similar was observed by [73] in Minnesota who proposed two explanations for this phenomenon: (1) similar geologic and topographical settings that favour sinkhole formation in areas of high concentration; or (2) changes in the hydraulic gradient around the existing sinkholes which increase solution and erosional processes that form more sinkholes. Considering that the majority of 1^st^ ranking sinkholes in Leskova dolina were located within a 300 meter wide elevation zone our results support the first explanation.

An interesting aspect from this study is the preferred directionality of the sinkhole orientation (Fig. 8). A first interpretation could be anisotropy in the autocorrelation of the error distribution of the lidar points’ position, which is sometimes referred to as “striping artefacts”, and was investigated by [74]. This interpretation has been discarded for the following two reasons: (i) when present, this error is evident in the hill-shade model but not in our case and (ii) the main orientation is neither parallel nor orthogonal to the flight direction. Another aspect that could add artificial bias to the sinkhole orientation is the geometry of the laser beam—surface interaction (incidence angle), which increases the position error in the component direction of the laser’s projection on the surface. However, this bias did not occur because orientation was not spatially correlated. If it were, we would have observed a preferred orientation in a specific position relative to the incidence angle between the terrain and lidar beam. Our findings regarding sinkhole orientation are also consistent with results, shown for dolines morphometric analysis and karst morphology of Biokovo Mountain [75].

In their recent review [1] point out that a comprehensive cartographic sinkhole inventory including morphometric parameters is the most important step in sinkhole hazard analysis. Such information is useful in risk assessment for new infrastructure construction [3], karst-related groundwater contamination and subsidence [76]. On the other hand, in unpopulated forested areas such as Leskova dolina a robust and semi-automatic method for mapping sinkholes from aerial lidar data can be very advantageous for planning access strategies in forests, especially as a previous study in Leskova dolina has shown greater tree growth inside the sinkholes due to deeper soil [45]. Because it is beneficial for forest activities to involve economic capitalisation, this aspect must be considered because it could be a factor that affects the overall values of forest areas.

Our investigations of the plant communities showed that the species richness of vascular plants was higher in the sinkholes relative to the surrounding area. Previous studies have indicated differences in vegetation compositions between sinkholes and their surrounding areas [33] where the sinkholes acted as refugia for glacial relicts, mountain species and wet-woodland plant species [37]. The roles of these refuge areas will be highlighted in view of predicted climatic changes. The climate-change predictions indicated that Dinaric fir-beech forests are the most threatened forest community in Slovenia [77]. In addition to their significant ecological and natural conservation roles, Dinaric fir-beech forests are among the most important forests for timber production. The affects of climate-changes on Dinaric fir-beech forests in this sensitive karst area are also associated with the distributions of sinkholes and other karst terrain characteristics, which could significantly change the frequency and severity of drought events and water drainage phenomenon. Considering the differences in sinkhole density in our study area, the use of lidar for detecting areas with high sinkhole densities could enable rapid designation of priority areas for conservation.

Overall, the non-subjective, fast and automated procedure for detection of sinkholes under forest canopies described in our case study could be used for many different purposes. This investigation led to ideas for future studies. For example, is the forest tree composition and/or structure spatially correlated with the sinkhole distribution? How topography influences spatial pattern of tree mortality, canopy decline and regeneration of Dinaric silver fir—European beech forests, as was studied by [78] and [79]. Continuations of this study could use classic remote sensing techniques (e.g., hyperspectral remote sensing data) with lidar data to assess such correlations. The use of full-waveform data can be leveraged to determine if an increase in the number of detected ground points results in significant improvement in sinkhole detection and characterisation. Full-waveforms require longer computation times and more processing steps [21], but are useful because they increase the number of return points and improve the ground point density [80].
